# Design and Validation of a Questionnaire to Measure the Perception of Nursing Degree Students about the Learning Process in Primary Care

**DOI:** 10.3390/healthcare10112240

**Published:** 2022-11-09

**Authors:** Adrián Almazor-Sirvent, Maria Dolores Miguel-Ruiz, Anna Huguet-Miguel, Bárbara Hurtado-Pardos, Juan Francisco Roldán-Merino, María del Carmen Moreno-Arroyo, Ainoa Biurrún-Garrido, Manuel Vicente Garnacho-Castaño, Anna Escofet

**Affiliations:** 1 Campus Docent Sant Joan de Déu, Universitat de Barcelona, 08830 Sant Boi de Llobregat, Barcelona, Spain; 2 Fundació Sant Joan de Déu, 08950 Esplugues del Llobregat, Barcelona, Spain; 3 Escola d’Infermeria Campus Bellvitge, Universitat de Barcelona, 08907 L’Hospitalet de Llobregat, Barcelona, Spain; 4 Facultat d’Educació, Universitat de Barcelona, 08035 Barcelona, Barcelona, Spain

**Keywords:** clinical practicum, reliability, instrument, perception of learning, nursing student competencies

## Abstract

The aim of this study was to develop a tool for the evaluation of the learning process of the clinical practicum in primary care. The study was carried out in two phases: (1) identification of the categories that determine the perception of the nursing degree students about the learning process in the clinical practicum in primary care and the items for each category; and (2) cross-sectional study in a sample of 475 nursing degree students. The psychometric properties in terms of reliability (internal consistency) and construct validity were analyzed through a confirmatory factor analysis. Cronbach’s alpha coefficient of internal consistency for the entire questionnaire was 0.93, and that for each of the categories was above 0.70 in all cases. The chi-squared test was statistically significant (2.84; *p* < 0.001). The confirmatory factor analysis produced a model of 6 dimensions and 41 items. The parameters were estimated through the least squares method. All saturations were statistically significant (*p* < 0.05). In view of the results of this study, it can be asserted that the questionnaire to measure the perception of the nursing degree students about the learning process in the community clinical practicum (QPCLP) presents good properties in terms of internal consistency and validity.

## 1. Introduction

Training related to the care context in the clinical practicum is fundamental in the learning process of nursing degree students [1] to acquire a set of general and specific competencies required for the adequate performance of their future profession [2].

Specifically, in the clinical practicum in primary care, new learning goals are established, which are aimed at both the promotion of health and the prevention of disease, focusing care not only on the person, but also on the family and the community. The nursing care provided from the community scope determines the perception of the students toward their own learning [3,4]. In this sense, this perception is influenced by different dimensions: the tutorship conducted by the practicum nurse and the academic tutor, the role adopted by the student, the theoretical subjects related to the clinical practicum, the learning environment generated, and the valuation of the clinical practicum in the community scope [5,6,7,8]. In this regard, there are learning needs inherent to the practicum of the nursing degree students in the scope of primary care. In community care, training is based on a perspective of care focused on the patient and the community, and not only on the absence of disease [9].

The instruments for the evaluation of the learning process are fundamental to measure the level of relevance of the clinical practicum in nursing degree students. These instruments must be complete and gather all the dimensions that intervene in each of the different periods of the practicum [10]. Numerous instruments have been used to evaluate the clinical learning process of nursing students. The Clinical Learning Environment scale (CLE-1995) analyzes the student-nursing team relationship, the commitment of the practicum tutor, the relationship with the patients, the interpersonal relationship, and student satisfaction [11]. Saarikoski and Leino-Kilpi (2002) developed the Clinical Learning Environment and Supervision scale (CLES-2002), whose aim is established in six factors: learning scope, supervisor leadership, service environment, characteristics of care, characteristics of learning, and relationship with the tutor [12]. In the scope of clinical simulation, Farrés-Tarafa et al. (2021) have recently developed an adaptation for Spanish nursing students, by validating the Student Satisfaction and Self-Confidence in Learning Scale (SCLS) [13]. The authors concluded that clinical simulations help the students to increase their levels of confidence and satisfaction, allowing them to face real scenarios in the clinical practice. Other studies have justified the importance of commitment, perception and practical training based on the evidence on Spanish nursing students [14]. 

The instruments analyzed in these studies were founded on the identification of the main factors that influence the perception of nursing students in the clinical practicum and clinical simulation, as well as the opinion of experts. However, no study has evaluated the nursing students’ perception toward the clinical practicum in the scope of primary care in the Spanish context. Moreover, these scales do not contextualize the learning process according to the type of specialized care offered by the practicum center. Therefore, it is necessary to create new instruments that consider the perception of students and graduates in Primary Care who have carried out the clinical practicum in primary care centers. This information could be very valuable through their experiences and their perception toward all those factors that influence the learning process, especially in a primary care environment, since such experiences allow for learning situations that differ from those encounters in the rest of the clinical practicum of the nursing degree. To the best our knowledge, there is no validated and reliable questionnaire to assess the perception of students toward their own learning in the context of Spanish primary care. 

The aim of this study was to design and validate a scale that measures, quantitatively and qualitatively, the perception of the students toward the learning process in the clinical practicum.

## 2. Materials and Methods

### 2.1. Design

Cross-sectional study conducted in two phases.

#### 2.1.1. Phase 1: Development of the Instrument

This phase was developed in four stages. In the first stage, a literature review was performed to identify the most determining dimensions described to date about the perception of nursing students toward the learning process in the clinical practicum. A total of six dimensions were identified: D1 (tutorship conducted by the practicum nurse), D2 (tutorship conducted by the academic tutor), D3 (student nursing performance), D4 (influence of the theoretical subjects directly related to the clinical practicum), D5 (learning environment generated during the clinical practicum), and D6 (valuation of the clinical practicum in the community scope). In the second stage, three work groups were established, each with 10 nursing students who had carried out the clinical practicum in the community scope, with the aim of identifying the possible items that facilitate or hinder the learning process in each of the described dimensions. The identification of the possible items that influence the learning of the clinical practicum was based on the consensus of the participants [15].

The first group identified a total of 20 items, whereas the second group identified 18 items, and the third group obtained a total of 22 items for all six dimensions. Then, to avoid duplicated items, a single list was created from the items provided by each group. The final list consisted of 54 items. 

In the third stage, a panel of 8 experts determined the content validity. Four expert professors participated in the tutoring process of the clinical practicum of different universities, as well as 4 nurses specialized in community health, with over 5 years of professional experience. Each expert valued each item according to the relevance in a scale of 1 (irrelevant) to 4 (very relevant). The content validity was calculated, for each item, as the percentage of experts that gave a score of 3 or 4. Only those items with a content validity index of 0.80 or higher were selected [16].

Of the 54 items, only 41 were evaluated as relevant or very relevant by the group of experts, thus representing match values of 0.80 or higher. 

Lastly, in the fourth stage, a pilot test was carried out in a group of 10 students to assess the completion time, clarity, and understandability of the different items. All students concluded that the questionnaire could be completed easily and quickly (10–15 min). After the debriefing, it was not necessary to modify the design or the content of the questionnaire. 

The instrument was called the nursing students’ perception of learning in the community clinical practicum (QPCLP).

#### 2.1.2. Phase 2: Validation of the Psychometric Properties of the QPCLP

Psychometric study of the reliability and validity of the QPCLP performed in a simple sample of 475 nursing students. 

### 2.2. Participants and Setting

The metric properties of the questionnaire were analyzed in a simple sample of 475 nursing students registered in the academic year 2018–2019 at Campus Docent Sant Joan de Déu and the University School of Nursing at the University of Barcelona. A non-probabilistic convenience sampling was applied. The study included students who had carried out their practicums in the community scope, excluding only those students who were not present when the questionnaire was administered. 

The sample size was calculated based on the recommendations of Streiner, Norman and Cairney (2015), who consider a sample of 5–20 participants for each item of the questionnaire [17]. For this study, it was decided to include a minimum of 10 students per item, resulting in a sample size of at least 410 participants. Comrey and Lee (1992) suggest a graduate scale to determine the sample size: 100 = poor, 200 = fair, 300 = good, 500 = very good, and 1000 = excellent [18]. Finally, a total of 475 students participated in the study.

The study was approved by the Research Ethics Committee of the Sant Joan de Déu Research Foundation CEIC PIC—106-18. All participants were informed about the goals of the study and the anonymity of all the data provided. 

### 2.3. Variables and Source of Information

A form with two sections was designed. The Section 1 is focused on sociodemographic (age, sex, access to university and employment), whereas the Section 2 includes the QPCLP, with its 41 items grouped in 6 dimensions. Each item is assessed in a scale of 1 to 4 points, based on the respondent’s degree of agreement with the items (1 = totally disagree; 2 = disagree; 3 = agree; 4 = totally agree). The sum of all the scores of each item allows obtaining a single measurement value that indicates the quantitative opinion of the students about the learning process of the clinical practicum; moreover, it also allows obtaining partial scores for each dimension of the questionnaire. The distribution of the items and the minimum and maximum scores of each dimension of the final questionnaire are shown in Table 1.

### 2.4. Statistical Analysis

The data were analyzed using SPSS statistical software (SPSS v22; Inc., Chicago, IL, USA), and the confirmatory factor analysis was conducted with EQS structural equations software (EQS 6.1 for Windows, Multivariate Software, Inc., Encino, CA, USA).

To analyze the internal consistency, Cronbach’s alpha was calculated, considering values between 0.70 and 0.90 to be acceptable [19]. 

Test-retest reliability was examined within a 4-week time frame using the intraclass correlation coefficient (ICC) criteria (two-factor and mixed-effects model), considering values between 0.70 and 0.90 to be acceptable [20].

Convergent and discriminant validity was evaluated through Spearman’s correlation coefficient between the total score of the questionnaire and the total scores of each of the dimensions. This analysis is based on the hypothesis that the correlation between each dimension and the questionnaire in general should be stronger than that which exists between dimensions [21].

To analyze the construct validity, a confirmatory factor analysis was carried out using the least squares method. A model of 6 dimensions was proposed, and the following fit indices were calculated: normalized chi-squared, defined as the ratio between the value of the chi-squared and the number of degrees of freedom (χ^2^/df); Goodness of Fit Index (GFI); Adjusted Goodness of Fit Index (AGFI); Comparative Fit Index (CFI); Bentler Bonnet Normed Fit Index (BNNFI); Bentler Bonnet Non-Normed Fit Index (BBNNFI); and Root Mean Standard Error of Approximation (RMSEA). The following values were considered as a good fit for the model: χ^2^/df values between 2 and 6 [22]; GFI, AGFI, CFI, BBNFI, and BBNNFI values ≥ 0.95 and RMSEA ≤ 0.05 [23,24]. CFA models were estimated using structural equation modelling (EQS 6.3 for Windows, Multivariate Software, Inc., Encino, CA, USA).

## 3. Results

### 3.1. Demographic Characteristics

Sociodemographic characteristics of the study population are presented in Table 2. The study included a total of 475 nursing students who had completed their nursing practicum in the community scope. The mean age was 23.4 years (SD 5.2), and most of the participants were women (84.2%). Regarding the university of origin, 52.6% were from the Nursing School of the Campus Sant Joan de Déu University of Barcelona. Most of the participants accessed university from baccalaureate (65.1%), followed by vocational training (25.9%). Of all the participating students, half of them were employed (56.2%) and worked an average of 23.0 h per week (SD 11.5), of whom 56.9% worked in healthcare.

### 3.2. Reliability

Cronbach’s alpha coefficient of internal consistency for the entire questionnaire was 0.93, and that for each of the categories was above 0.70 in all cases. The alpha values were also calculated excluding each item from the questionnaire, observing that such item removal did not improve the internal consistency in a relevant manner in any case (Table 3).

The ICC analysis demonstrated that the 4-week test-retest reliability was 0.72 (95% confidence interval 0.56–0.828, *n* = 475) and was satisfactory for the six subscales or dimensions (Table 4).

### 3.3. Convergent and Discriminant Validity

With respect to the analysis of the correlations between the subscales, the strongest correlations were obtained between the subscales and the total scale. Dimension D1 (tutorship conducted by the practicum nurse) presented the strongest correlation with the total scale (rho = 0.747), whereas dimension D4 (influence of the theoretical subjects directly related to the clinical practicum) showed the weakest correlation with the total scale (rho = 0.606). Between the subscales, the strongest correlation was obtained between D1 (tutorship conducted by the practicum nurse) and D5 (learning environment generated during the clinical practicum) (rho = 0.642), whereas the weakest correlation was identified between dimension D1 (tutorship conducted by the practicum nurse) and D4 (influence of the theoretical subjects directly related to the clinical practicum) (rho = 0.264) (Table 5). All correlations were statistically significant (*p* < 0.01).

### 3.4. Construct Validity

Dimensions D1 and D2 showed the greatest factor loading or saturation. All saturations were above 0.5, except for item 2 (0.318), item 20 (0.482) and item 32 (0.441) (Table 6). 

The chi-squared test was statistically significant (*p* < 0.001), with a fit ratio of 2.84. Likewise, the rest of the analyzed indices presented adequate values of the fitted model (Table 7).

## 4. Discussion

The QPCLP is a psychometric tool, composed of 41 items and 6 dimensions, that can be easily used and administered. From the 41 items identified, a questionnaire was developed to determine the perception of nursing students toward their learning in the community clinical practicum. Although it was not analyzed in this study, the data established in phase 1 was a key factor in the development of the QPCLP [25].

The psychometric characteristics that were obtained from the questionnaire are adequately fitted to each of the dimensions. The developed dimensions showed high internal consistency in relation to the total questionnaire (0.93), taking into account that, in the construction of a measurement instrument, the minimum acceptable reliability must be 0.70 [19,26]. Except for category 3 (student nursing performance), which obtained an internal consistency of 0.70, all the other categories presented an internal consistency higher than 0.70. Other authors suggest values above 0.60 to consider an acceptable reliability value of the measurement [27]. The internal consistency reported was high, in line with that of other studies that have also proposed the development of this type of tools related to the clinical practicum of clinical nursing students [11,12,28,29,30]. The corrected alpha coefficient showed that the removal of any of the items did not increase the internal consistency of the questionnaire. Therefore, excluding any of the items from the scale could be unwise [28].

Regarding the convergent and discriminant validity, all dimensions are consistent with respect to the total scale, although the subscales show some weaker yet significant correlations between the different dimensions. 

In regard with the construct validity, the confirmatory factor analysis showed that D1 and D2 obtained the greatest loading factors, with all of them being above 0.5, except for 3 items. This indicates the adequate purpose of the tool and the nature of the standards of the nursing students in the community practicum. To determine that this factor structure is compatible and demonstrates the validation of the construct, it is strongly recommended to perform confirmatory factor analyses [31,32].

The analysis of the dimensions confirmed that the QPCLP consistently reflected the construct for which it was design, i.e., to measure the perception of nursing students toward the community clinical practicum. The main limitation in the development of a construct is specifying the number of dimensions that define it according to Cronbach [33]. From this point, we believe that the factor structure of the QPCLP allows defining and assessing the construct “perception of learning” through six dimensions that are satisfactorily correlated to each other and do not surpass the correlation with the total scale in any case.

The parameters were estimated using the least squares method. This method is usually applied for items of ordinal measuring, and it has the same properties as the maximum likelihood method, although under less rigorous considerations of multivariate normality [34]. The fit ratio for the model was statistically significant (2.84), thus it is in the range of 2–6, i.e., the fit is reasonably good [34]. Similarly, the rest of the analyzed indices present the same tendency; therefore, it can be concluded that the model fits adequately. The CFI shows a value of 0.95, indicating that the model can be considered acceptable, as it is above 0.90 [22]. Likewise, the RMSEA obtained a value of 0.039, which is below 0.060, thereby indicating that the model is adequate [22]. 

In the year 2020, the Research Priorities Subcommittee of the Association of Nursing Students of Public/Community Healthcare (ACHNEI in Spanish), published a report on the state of education in public healthcare nursing, which highlights the need for evidence on the impact of public/community healthcare nursing teaching on communities and students [35]. 

This report proposes an action research model as a means for the continuous advance of this discipline. Thus, regarding the training of nursing students, focusing on the practicum carried out by the nursing degree students in the scope of primary care, it is observed that there are learning needs inherent to this training period, since, in community care, training is based on a perspective of care centered on the patient and on the community, and not only on the absence of disease [9,36]. 

In addition to the above mentioned, the literature points out the need for rigorous scientific studies that underline the impact and efficacy of education in public and community healthcare. Moreover, no studies have assessed the perception of students toward their own learning in the Spanish context. Therefore, it is important to generate valid and reliable instruments that measure the perception of nursing degree students about the learning process of the community clinical practicum. 

The values obtained in the evaluation of the reliability and validity of the questionnaire justify the creation of this type of questionnaires, given the lack of tools that evaluate the clinical practicum of community nursing students, considering the differences between the hospital and community scopes. 

Several limitations must be considered. The study population was exclusively focused on students of Sant Joan de Déu and the University Nursing School of the University of Barcelona. However, since other faculties and university nursing schools of Spain have similar study plans in relation to the clinical practicum in primary care, the results can be extrapolated. In addition, the high percentage of women in the study (84.9%) shows that there is no gender-equitable sample. No questionnaire from other studies was used as the gold standard, therefore, future studies should analyze the convergent validity with other similar instruments that measure the same construct or study phenomenon.

## 5. Conclusions

The QPCLP presents good psychometric properties in terms of internal consistency and validity, and it measures six dimensions: tutorship of the nursing professional, tutorship of the practicum tutor, the student nursing performance, theoretical subjects related to the clinical practice, the learning environment, and evaluation of the clinical practicum. 

The creation of this type of tools is essential, given the lack of instruments that assess the clinical practicum of nursing students in the community scope. 

## Figures and Tables

**Table 1:** Distribution, by category, of the evaluation questionnaire items for the learning of the clinical practicum, and minimum and maximum values for each item and for the questionnaire overall.

Dimensions	Items	Minimum Score	Maximum Score
D1: Tutorship conducted by the practicum nurse	From 1 to 9	9	36
D2: Tutorship conducted by the academic tutor	From 10 to 17	8	32
D3: Student nursing performance	From 18 to 22	5	20
D4: Influence of the theoretical subjects directly related to the clinical practicum	From 23 to 27	5	20
D5: Learning environment generated during the clinical practicum	From 28 to 34	7	28
D6: Valuation of the clinical practicum in the community scope	From 35 to 41	7	28
Total	From 1 to 41	41	164

**Table 2:** Sociodemographic characteristics of the study population (*n* = 475).

	*n*	%
Age mean (SD)	23.4 (5.2)	
Sex		
Men	75	15.8
Women	400	84.2
University of origin		
Campus Docent SJD	250	52.6
Campus Bellvitge	225	47.4
Accessed university		
baccalaureate	309	65.1
vocational training	123	25.9
Other university studies	16	3.4
Others	27	5.7
Employed		
Yes	267	56.2
No	208	43.8
Worked an average (SD)	23.0 (11.5)	
Worked in healthcare		
Yes	152	56.9
No	115	43.1

**Table 3:** Internal consistency coefficient (Cronbach’s alpha) of the Questionnaire of Perception toward the Clinical Learning Process (QPCLP).

Summarized Content of the Items		Cronbach’s Alpha		
		Total Subscale	Total Subscale Sin Ítem	Total Scale Sin Ítem
D1. Tutorship conducted by the practicum nurse		0.880		
1.	During the community practicum, my reference nurse facilitated my learning		0.859	0.936
2.	Having different reference nurses during the practicum can be a learning advantage		0.905	0.939
3.	My reference nurse integrated me in his/her daily tasks		0.860	0.936
4.	My reference nurse knew how to transmit his/her knowledge thanks to his/her expertise		0.862	0.936
5.	My reference nurse favored my participation in decision making		0.865	0.937
6.	The experience of my reference nurse posed an added value for my learning process		0.860	0.936
7.	Establishing friendship bonds with my reference nurse made me feel more confident in decision making		0.862	0.936
8.	My reference nurse made me reflect on my actions during the practicum		0.868	0.936
9.	My reference nurse generated a climate of trust during the teaching-learning process		0.856	0.936
D2. Tutorship conducted by the academic tutor		0.904		
10.	I consider that the physical presence of my tutor is important for the follow-up of my clinical learning		0.914	0.938
11.	My practicum tutor was reachable		0.885	0.937
12.	My practicum tutor was flexible		0.887	0.937
13.	My tutor guided me in my learning process during the clinical practicum		0.880	0.937
14.	My tutor showed interest and helped me to solve problems		0.879	0.936
15.	I consider that my tutor was fair in the application of the regulations of the clinical practicum		0.888	0.936
16.	My tutor facilitated my learning		0.879	0.937
17.	I consider that the practicum tutor must know how the university works		0.914	0.938
D3: Student nursing performance		0.708		
18.	The management of my emotions during the practicum enabled a meaningful learning		0.636	0.937
19.	I consider that I was motivated during the practicum		0.656	0.936
20.	My previous theoretical knowledge allowed me to support the clinical practicum		0.709	0.937
21.	My own personality helped me to learn		0.645	0.937
22.	I reflected on my actions during the practicum		0.652	0.937
D4: Influence of the theoretical subjects directly related to the clinical practicum practicum		0.855		
23.	The subject Community Nursing helped me to support the clinical practicum in primary care		0.741	0.938
24.	The organization of the subject Community Nursing allowed me to acquire the competencies associated with the clinical practicum in primary care		0.749	0.938
25.	I remembered all the theoretical contents at the beginning of the practicum		0.790	0.938
26.	I consider that the teaching methodologies based on the practicum improve my learning		0.820	0.938
27.	I consider all the subjects related to primary care to be useful for my performance in the clinical practicum in primary care		0.764	0.937
D5. Learning environment		0.828		
28.	My learning in the clinical practicum developed in an environment of trust and respect		0.789	0.936
29.	I felt included in the team during the clinical practicum		0.793	0.936
30.	The climate I perceived during the practicum enabled my academic and personal development		0.783	0.936
31.	The contract situation (permanent, temporary or substitute) of the nurses at the primary care centre favoured the teaching and learning process		0.827	0.937
32.	Once I knew the environment in which the clinical practicum takes place in primary care, I identified my near professional future within this scope		0.860	0.938
33.	I consider that, during the practicum, there was a climate based on companionship		0.783	0.936
34.	I consider that, during the practicum, there was an atmosphere based on team work		0.796	0.937
D6. Valuation of the clinical practicum in primary care		0.882		
35.	I consider that I learned theoretical knowledge related to primary care		0.864	0.936
36.	I consider that I learned practical skills related to primary care		0.853	0.936
37.	I consider that I learned attitudes related to primary care		0.859	0.936
38.	I recognise that the nursing role in primary care is very relevant		0.859	0.937
39.	My perception toward the work of the primary care professionals was better after the practicum		0.862	0.937
40.	I consider that the nursing work in primary care is different from that in the hospital scope		0.886	0.938
41.	I would recommend the clinical practicum in primary care to other students		0.873	0.936

**Table 4:** Test-retest Intraclass Correlation Coefficient (ICC) of the Questionnaire of Perception toward the Clinical Learning Process (QPCLP).

Categories	Intraclass Correlation Coefficient (ICC)	Confidence Interval (95% CI)
Category 1. Tutorship of the nursing professional	0.702	0.529–0.812
Category 2. Tutorship of the practicum	0.702	0.529–0.812
Category 3. Student nursing performance	0.665	0.469–0.788
Category 4. Theoretical subjects related to the clinical practicum	0.999	0.999–0.999
Category 5. Learning environment	0.758	0.618–0.847
Category 6. Valuation of the clinical practicum in primary care	0.720	0.557–0.823
TOTAL	0.728	0.569–0.828

**Table 5:** Correlations of the Questionnaire of Perception toward the Clinical Learning Process (QPCLP) and total questionnaire (rho).

	D1	D2	D3	D4	D5	D6
D1: Tutorship conducted by the nursing professional	1					
D2: Tutorship conducted by the academic tutor	0.390 *	1				
D3: Student nursing performance	0.493 *	0.344 *	1			
D4: Influence of the subjects directly related to the clinical practicum	0.264 *	0.368 *	0.449 *	1		
D5: Learning environment generated during the clinical practicum	0.642 *	0.329 *	0.484 *	0.327 *	1	
D6: Valuation of the clinical practicum in the community scope	0.479 *	0.301 *	0.489 *	0.333 *	0.531 *	1
TOTAL	0.747 *	0.738 *	0.671 *	0.606 *	0.737 *	0.644 *

* All correlations are significant; significance level *p* < 0.01.

**Table 6:** Factor loadings derived from the LS (least squared) estimation of the Confirmatory Factor Analysis (λij).

Item	Factor 1	Factor 2	Factor 3	Factor 4	Factor 5	Factor 6
1	0.774 *					
2	0.318 *					
3	0.782 *					
4	0.710 *					
5	0.678 *					
6	0.778 *					
7	0.730 *					
8	0.666 *					
9	0.786 *					
10		0.509 *				
11		0.737 *				
12		0.788 *				
13		0.797 *				
14		0.857 *				
15		0.853 *				
16		0.825 *				
17		0.507 *				
18			0.603 *			
19			0.698 *			
20			0.482 *			
21			0.549 *			
22			0.559 *			
23				0.738 *		
24				0.624 *		
25				0.571 *		
26				0.687 *		
27				0.753 *		
28					0.774 *	
29					0.760 *	
30					0.821 *	
31					0.527 *	
32					0.441 *	
33					0.731 *	
34					0.662 *	
35						0.747 *
36						0.796 *
37						0.772 *
38						0.730 *
39						0.729 *
40						0.532 *
41						0.753 *

*All correlations are significant; significance level *p* < 0.01.

**Table 7:** Goodness of fit indices of the Confirmatory Model.

INDEX	VALUE
BBNFI	0.887
BBNNFI	0.946
GFI	0.973
AGFI	0.970
CFI	0.949
RMSE	0.057
RMSEA	0.039
Cronbach’s α	0.943
Goodness of fit test	χ^2^ = 2171.058; gl = 764; *p* = 0.000
Fit ratio	χ^2^/gL = 2.84 entre 2–6

BBNFI: Bentler Bonnet Normed Fit Index. BBNNFI: Bentler Bonnet Non-Normed Fit Index. GFI: Goodness of Fit Index. AGFI: Adjusted Goodnes. CFI: Comparative Fit Index. RMSE: Root Mean Standard Error. RMSEA: Root Mean Standard Error Approximation.

## Data Availability

The data presented in this study are available upon request from the authors. Some variables were restricted to preserve the anonymity of study participants.
